# Mobile Phone-Based Telemonitoring for Heart Failure Management: A Randomized Controlled Trial

**DOI:** 10.2196/jmir.1909

**Published:** 2012-02-16

**Authors:** Emily Seto, Kevin J Leonard, Joseph A Cafazzo, Jan Barnsley, Caterina Masino, Heather J Ross

**Affiliations:** ^1^Centre for Global eHealth InnovationUniversity Health NetworkToronto, ONCanada; ^2^Department of Health Policy, Management and EvaluationUniversity of TorontoToronto, ONCanada; ^3^Institute of Biomaterials and Biomedical EngineeringUniversity of TorontoToronto, ONCanada; ^4^Department of MedicineUniversity of TorontoToronto, ONCanada; ^5^Divisions of Cardiology and TransplantUniversity Health NetworkToronto, ONCanada

**Keywords:** heart failure, telemedicine, mobile phone, patient monitoring, randomized controlled trial

## Abstract

**Background:**

Previous trials of telemonitoring for heart failure management have reported inconsistent results, largely due to diverse intervention and study designs. Mobile phones are becoming ubiquitous and economical, but the feasibility and efficacy of a mobile phone-based telemonitoring system have not been determined.

**Objective:**

The objective of this trial was to investigate the effects of a mobile phone-based telemonitoring system on heart failure management and outcomes.

**Methods:**

One hundred patients were recruited from a heart function clinic and randomized into telemonitoring and control groups. The telemonitoring group (N = 50) took daily weight and blood pressure readings and weekly single-lead ECGs, and answered daily symptom questions on a mobile phone over 6 months. Readings were automatically transmitted wirelessly to the mobile phone and then to data servers. Instructions were sent to the patients’ mobile phones and alerts to a cardiologist’s mobile phone as required.

**Results:**

Baseline questionnaires were completed and returned by 94 patients, and 84 patients returned post-study questionnaires. About 70% of telemonitoring patients completed at least 80% of their possible daily readings. The change in quality of life from baseline to post-study, as measured with the Minnesota Living with Heart Failure Questionnaire, was significantly greater for the telemonitoring group compared to the control group (*P* = .05). A between-group analysis also found greater post-study self-care maintenance (measured with the Self-Care of Heart Failure Index) for the telemonitoring group (*P* = .03). Brain natriuretic peptide (BNP) levels, self-care management, and left ventricular ejection fraction (LVEF) improved significantly for both groups from baseline to post-study, but did not show a between-group difference. However, a subgroup within-group analysis using the data from the 63 patients who had attended the heart function clinic for more than 6 months revealed the telemonitoring group had significant improvements from baseline to post-study in BNP (decreased by 150 pg/mL, *P* = .02), LVEF (increased by 7.4%, *P* = .005) and self-care maintenance (increased by 7 points, *P* = .05) and management (increased by 14 points, *P* = .03), while the control group did not. No differences were found between the telemonitoring and control groups in terms of hospitalization, mortality, or emergency department visits, but the trial was underpowered to detect differences in these metrics.

**Conclusions:**

Our findings provide evidence of improved quality of life through improved self-care and clinical management from a mobile phone-based telemonitoring system. The use of the mobile phone-based system had high adherence and was feasible for patients, including the elderly and those with no experience with mobile phones.

**Trial Registration:**

ClinicalTrials.gov NCT00778986

## Introduction

The demand for health care resources to manage heart failure is increasing with the aging population. Innovative methods to help alleviate this burden and to improve the poor outcomes from heart failure are required. Previous studies on traditional telemonitoring of heart failure patients (ie, using dedicated hardware as an information and transmission hub) have determined telemonitoring has the potential to reduce mortality, hospitalizations, and costs as well as improve quality of life, self-care, and New York Heart Association (NYHA) class [1-4]. However, the results of telemonitoring trials have been inconsistent largely due to diverse study interventions and variations in study design.

Little is known about the feasibility and effects of mobile phone-based telemonitoring systems. Investigating mobile systems is a logical next step because they enable greater scalability of telemonitoring due to their relatively low cost compared to traditional systems, and because they provide more freedom for the patient due to their portability. Two recent randomized controlled trials of mobile phone-based telemonitoring for heart failure management have been reported in literature [5,6]. The Telemedical Interventional Monitoring in Heart Failure (TIM-HF) trial by Koehler et al (2011) found no reductions in hospitalizations or mortality [5]. The authors concluded their study does not rule out the potential benefits of telemonitoring, but instead that there is a need to identify the heart failure population that could benefit from telemonitoring [5]. The trial by Scherr et al (2009), which required patients to enter readings using a mobile phone’s Internet browser, highlighted the importance of system design on the success of the telemonitoring system [6]. Many patients found using the Internet browser to be too difficult, resulting in 12 out of the 54 patients in the intervention group being “never beginners.”

The objective of our randomized controlled trial was to perform an in-depth investigation of the effects of a highly automated and user-centered mobile phone-based telemonitoring system on self-care and clinical management, with the aim of improving heart failure outcomes (Trial Registration: ClinicalTrials.gov NCT00778986). We used an extensive user-centered design process to develop the telemonitoring system in order to ensure it was as easy to use as possible and to meet the needs of the clinicians and patients. User-centered design refers to a philosophy that bases the design on information about and input from the people who will be using the product. The user-centered design process will be discussed in a separate publication.

## Methods

### Study Participants

**Figure 1:**
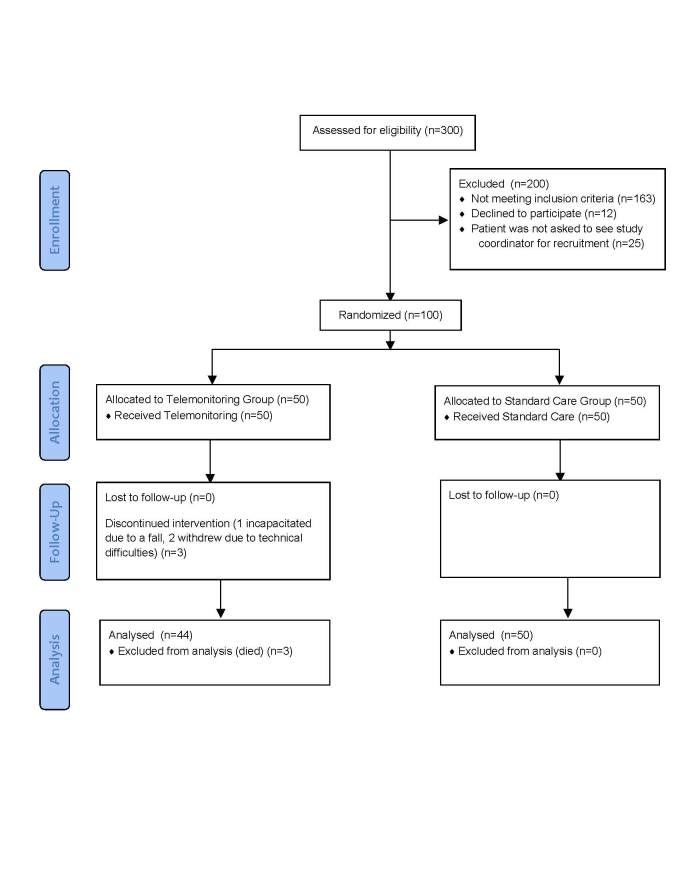
Flow of participants through the trial.

One hundred participants were recruited from the University Health Network (UHN) Heart Function Clinic in Toronto, Ontario, between September 2009 and February 2010 (Figure 1). The UHN Research Ethics Board approved the trial prior to commencement. The primary intent of the trial was to pilot the telemonitoring system in order to determine the impact of the system on self-care and clinical management. A sample size calculation was based on the Self-Care of Heart Failure Index (SCHFI), using a population standard deviation of 20 and an effect size of 10 (effect size represents a clinically significant change of more than half a standard deviation) as determined in previous studies (alpha = 0.05, power = 0.8) [7,8]. We calculated the required sample size per group to be 34, and recruited 50 participants for the intervention group and 50 for the control group to compensate for the patients estimated as lost to follow-up, including due to mortality, over the six-month trial.

Eligible participants were ambulatory patients diagnosed with heart failure. Other eligibility criteria included 18 years of age or older, ability to speak and read in English, not on the heart transplantation list, an expected survival of greater than one year, and a left ventricular ejection fraction (LVEF) less than 40%. During their Heart Function Clinic visit, patients who met the inclusion criteria (as deemed by their cardiologist), were invited to speak to the study coordinator (ES) regarding participation in the study. Each participant provided informed consent and received Can $24 as reimbursement for travel and parking expenses.

### Study Protocol

The 100 participants were randomized into the telemonitoring (TM) group and standard care (SC) group using stratified four-block randomization. Stratification was based on NYHA classification (NYHA class II-III and NYHA class IV). There were no participants in NYHA class I. An online computer-generated randomization tool, Research Randomizer [9], was used to determine the order of participants in the telemonitoring and standard care groups. The study coordinator was blinded to which group the patient would be assigned until each patient consented to participate in the trial.

Each patient received a questionnaire to complete at home. The questionnaire included demographic and clinical characteristic questions, and the SCHFI and the Minnesota Living with Heart Failure Questionnaire (MLHFQ), which are validated tools for measuring self-care and quality of life, respectively [10-12]. The SCHFI is made up of three subscales: self-care maintenance (choice of behaviors used to maintain physiological stability), self-care management (response to symptoms when they occur), and self-care confidence. The maintenance, management, and confidence subscales consist of 5, 6, and 4 Likert questions, respectively. A higher score on the SCHFI indicates improved self-care. The MLHFQ consists of 21 questions that use a 6-point Likert scale. The physical dimension score for the MLHFQ is the summation of 8 questions (eg, Did your heart failure make you sit or lie down to rest during the day?), while the emotional dimension score is the summation of 5 other questions (eg, Did your heart failure make you worry?). A lower score on the MLHFQ indicates higher quality of life.

The standard care group received standard care at the UHN Heart Function Clinic, which includes visiting the clinic between once every 2 weeks to once every 3 to 6 months, depending on the severity of the patient’s heart failure condition and the need for optimizing their medication. Standard care also includes heart failure education during preliminary visits at the Heart Function Clinic and the ability to telephone the clinic as necessary. Participants in the standard care group were not contacted again regarding the study until the end of the trial.

The participants in the telemonitoring group received the telemonitoring system in addition to standard care. They were asked to use the telemonitoring system for 6 months to take daily morning weight and blood pressure readings as well as weekly single-lead electrocardiograms (ECGs) if provided with an ECG recorder. They were also asked to answer daily morning symptom questions on a mobile phone. Only the 17 patients who did not have an implantable cardioverter defibrillator (ICD) were provided with an ECG recorder because the recorder was not certified for use with ICDs. Patients were also told to report their symptoms through the mobile phone if they did not feel well during the day. The patients in the telemonitoring group were given an individual training session on how to use the system during the recruitment session, and were provided with technical support by telephone throughout the study. The daily measurements took about 5 minutes each morning.

Six months after recruitment, all participants were mailed a post-study questionnaire. Semi-structured interviews were conducted with participants in the telemonitoring group to determine their experiences with the system. Twenty-two patients were interviewed, at which point saturation was achieved (ie, no new themes were identified). The individual interviews lasted between 15 and 60 minutes and took place in a private consultation room at the Heart Function Clinic.

The five clinicians (three cardiologists and two nurse practitioners) from the Heart Function Clinic who managed the alerts and/or used the data from the telemonitoring system during the trial were also interviewed post-study. Each semi-structured interview lasted between 15 and 45 minutes. All patient and clinician interviews were audio-recorded and later transcribed.

### Telemonitoring System Overview

**Figure 2:**
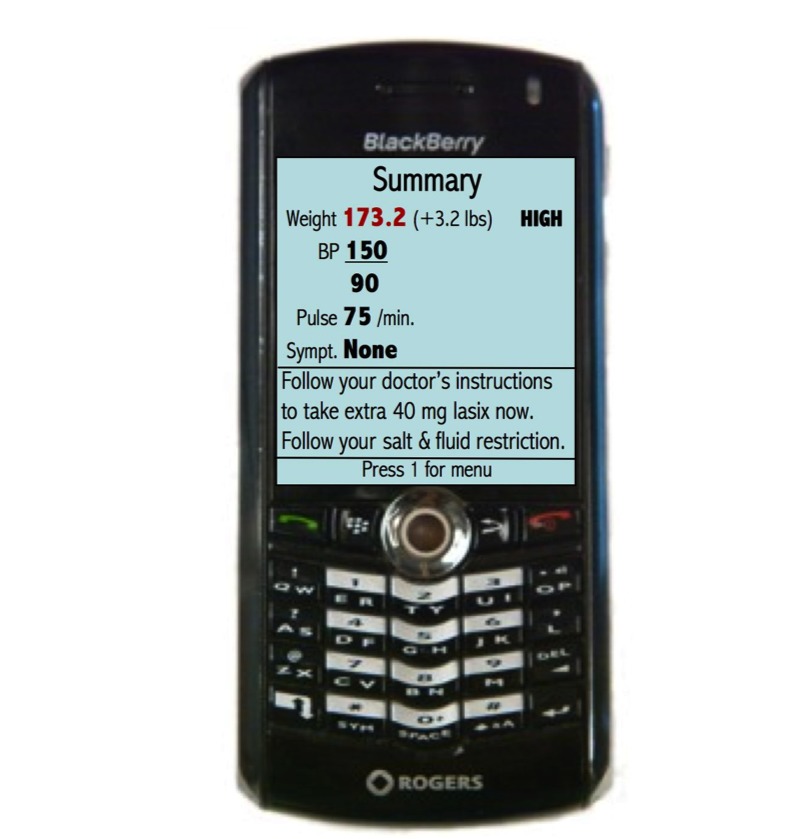
Sample message sent to patient’s mobile phone.

The weight and blood pressure readings (UA UC-321PBT weight scale and UA-767PBT blood pressure monitor, A&D Medical, USA) and ECG recordings (SelfCheck ECG PMP4, CardGuard, Israel) were automatically sent wirelessly via Bluetooth to a mobile phone (BlackBerry Pearl 8130, Research in Motion, Canada) and then to the data repository at the hospital. Patients also answered symptom questions (mainly yes/no) through the mobile phones. The measurements sent to the data repository included a study identifier. No patient names were transmitted and no identifying information was stored on the mobile phone. The mobile phone displayed an instruction on what to do after taking each measurement. A final message or alert based on the physiological and symptom information was sent to the mobile phone (Figure 2). Clinicians were able to modify any necessary physiological target ranges per patient through a secure website. The alerts ranged from low priority ones (eg, to retake the measurements if the patient feels worse) to high priority alerts (eg, to go to the local emergency department or call 911). The patients and the clinicians were able to view the physiological data on a secure website in tabular and graphic formats. All the data were also stored and accessible on the mobile phone. If a patient did not take all the required measurements by 10 am each morning, an automated adherence reminder phone call was sent to their home telephone.

If measurements were outside of the target range or if the patient reported symptoms, alerts were emailed to the cardiologist’s mobile phone. The email alerts included the patient’s contact information, medication list, symptom and physiological information (including the ECG recording as an attachment, if available), latest serum creatinine and potassium, and the alert message sent to the patient. If the cardiologist determined contacting the patient was warranted, they were able to call the patient by selecting their phone number in the email. The patient alert message also instructed them to contact the Heart Function Clinic if they felt they should. During the trial, the cardiologist usually called the patient within a few minutes of receiving the alert. The cardiologist emailed the study coordinator specifying the action resulting from each alert (eg, calling the patient, modificaton of medications, etc) by replying to the original email alert.

### Outcome Measures

The primary outcomes of this study included a surrogate for heart failure prognosis, specifically brain natriuretic peptide (BNP), self-care as measured by the SCHFI, and quality of life as measured by the MLHFQ. Hospital readmissions, number of nights in hospital, and mortality were secondary outcome measures because the study was underpowered to detect differences between groups for these metrics. Other secondary outcome measures included number of emergency department visits and number of Heart Function Clinic visits. In addition, LVEF, NYHA class, medication prescriptions, and blood test results (specifically creatinine, sodium, potassium, hemoglobin, and urate values) were also subsequently analyzed.

### Data Analysis

The normality of the data for each outcome measure was determined through Kolmogorov–Smirnov and Shapiro–Wilk tests of normality. Data that were normally distributed were MLHFQ, SCHFI maintenance, SCHFI management, sodium, potassium, hemoglobin, urate, and LVEF values. All other parameters were analyzed with non-parametric tests.

Between-group analyses using independent Student *t* tests and Mann–Whitney tests (for normally and not normally distributed data, respectively) were first performed to compare the telemonitoring group and standard care group post-study data. Between-group analyses were also performed to compare the change scores. Paired Student *t* tests and Wilcoxon signed rank tests were then performed to compare baseline and post-study data within the telemonitoring and standard care groups. The statistical analyses were performed using the statistical software application SPSS 17.0 (IBM Corporation, USA). Statistical significance was considered at *P* < .05 unless otherwise specified. All test results reported are 2-tailed. 

Interview data were analyzed using a conventional content analysis approach [13]. Two researchers (ES and CM) analyzed the transcripts independently and coded the transcripts with the software program NVivo version 7 (QSR International, Doncaster, Victoria, Australia). The researchers then discussed the themes and issues that emerged until a consensus was reached. The qualitative results are presented in detail in an accompanying paper [14].

## Results

Twelve out of 112 patients approached to participate in this study declined. One patient felt overwhelmed by the idea of participating in the trial, two did not want to take measurements every day, one said he did not have the time, and one thought as a result of a stroke he would not be able to understand how to perform the monitoring. The remainder did not provide specific reasons for declining. The three patients from the telemonitoring group who withdrew from the study included a patient who became incapacitated during the trial due to a fall, and two participants who decided to withdraw from the study when they had technical difficulties with the telemonitoring equipment. No patients in the standard care group officially withdrew from the study (Figure 1).

### Baseline Patient Data 

Table 1 shows the baseline demographic and clinical characteristics of the 100 patients who participated in the trial. The profiles of the telemonitoring and standard care groups were similar and representative of the patient population attending the UHN Heart Function Clinic. A comparison of baseline study parameters showed no statistical differences between telemonitoring and standard care groups for any outcome measures. Baseline questionnaires were completed and returned by 94 patients (46 from the telemonitoring group and 48 from the standard care group) and 84 patients returned post-study questionnaires (39 from the telemonitoring group and 45 from the standard care group), while 82 patients returned both baseline and post-study questionnaires.

### Telemonitoring System Utilization

#### Patient Adherence

**Figure 3:**
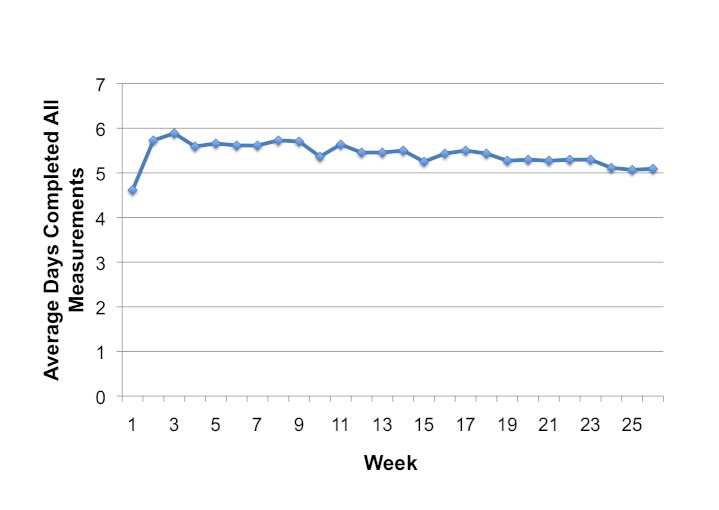
Weekly adherence to completing all daily measurements.

Patients completed their required measurements on average between 5 to 6 days per week throughout the six-month trial (Figure 3). Adherence decreased only slightly from the beginning of the trial to the end. Adherence during the first week was relatively low because some patients had to travel for a number of days to get home before using the system, and some patients required technical telephone support before properly using the system. About 42, 33, and 16 out of the 50 telemonitoring group patients (84%, 66%, and 32%) completed at least 91 (50%), 146 (80%), and 173 (95%) of possible daily readings over the six months, respectively. Missed measurements were sometimes due to technical issues with the telemonitoring system or else because of patients going on vacation without bringing the monitoring equipment with them.

The adherence data presented are an underestimate of the true adherence because they are based on the adherence phone calls sent at 10 am if patients had not yet completed their daily measurements. Occasionally, patients would take their measurements after 10 am when the adherence reminder was already sent because they had woken after 10 am.

#### Clinical Utilization of the System

Table 2 summarizes the clinical utilization of the system and the actions the clinicians performed based on the alerts. One cardiologist received the majority (1367) of the email alerts. Another cardiologist received alerts (311 alerts) when covering for the primary cardiologist for a three-week period. Low priority alerts were more frequently generated than the more urgent alerts. The cardiologists (and occasionally a nurse practitioner) called patients 480 times over the 6 months, often to provide instructions or to educate. The most common clinical action was an instruction or change in medication (105 times). Other actions included ordering additional blood work, moving the patient’s clinic visit forward, or instructing the patient to see their family physician or to go to the emergency department. 

**Table 1:** Baseline demographic and clinical characteristics of patient participants^a^

Characteristic		telemonitoring group (N = 50)	standard care group (N = 50)
**Age, mean (SD), years**		55.1 (13.7)	52.3 (13.7)
**Gender, No. (%)**	Male	41 (82)	38 (76)
	Female	9 (18)	12 (24)
**Ethnicity, No. (%)**	Caucasian	39 (78)	33 (66)
	African Canadian	5 (10)	4 (8)
	South East Asian	2 (4)	2 (4)
	Chinese	0	3 (6)
	Other	4 (8)	8 (16)
**Marital status, No. (%)**	Married	34 (68)	28 (56)
	Not Married	12 (24)	19 (38)
**Living arrangement, No. (%)**	Living with partner or family	42 (84)	38 (76)
	Living alone	6 (12)	9 (18)
**Highest education level achieved, No. (%)**	Less than high school	1 (2)	6 (12)
	High school	12 (24)	13 (26)
	College/University	33 (66)	28 (56)
**Income, No. (%)**	< $15,000	9 (18)	11 (22)
	$15,000-$49,999	16 (32)	18 (36)
	> $50,000	15 (30)	13 (26)
	Preferred not to answer	6 (12)	5 (10)
**Employment, No. (%)**	Full-time	13 (26)	14 (28)
	Part-time	3 (6)	1 (2)
	Disability due to heart failure	18 (36)	19 (38)
	Retired	8 (16)	7 (14)
	Unemployed	4 (8)	7 (14)
**NYHA class, No. (%)**	II	21 (42)	22 (44)
	II/III	6 (12)	5 (10)
	III	21 (42)	21 (42)
	IV	2 (4)	2 (4)
**Left ventricular ejection fraction, mean (SD)**		27.1 (7.8)	27.0 (9.9)
**Blood pressure, mean (SD)**	Systolic	108 (17)	102 (16)
	Diastolic	69 (13)	66 (11)
Pulse, mean (SD), beats/minute		73 (11)	73 (13)
**Blood test values, mean (SD)**	Creatinine, umol/L	108 (34)	105 (41)
	Sodium, mmol/L	139 (3)	139 (3)
	Potassium, mmol/L	4.4 (0.4)	4.2 (0.5)
	Hemoglobin, g/L	135 (13)	142 (15)
	Urate, umol/L	413 (117)	412 (124)

**Duration of heart failure, median, (interquartile range), years**		4.8 (7.8)	3.5 (8.2)
**Primary cause of heart failure, No. (%)**	Ischemic	20 (40)	13 (26)
	Idiopathic	22 (44)	29 (58)
	Other	8 (16)	8 (16)

^a^Missing values account for totals less than 100%.

**Table 2:** Clinical utilization of the telemonitoring system and clinical actions resulting from alerts

Clinical system use/action	Times occurred over the 6-month trial
Email alerts sent to Cardiologist A; Cardiologist B	1367; 311
Logged onto website by Cardiologist A; Nurse Practitioner A	67; 34
Phoned patient due to alerts	480
Medication changed or medication instructions given	105
Ordered additional blood work	26
Moved clinic visit forward to an earlier date	9
Instructed patient to go to local emergency department	6
Instructed patient to contact family physician	4

### BNP, NYHA Class, LVEF, SCHFI, and MLHFQ Results 

Table 3 shows the results from the statistical analysis. BNP values for both the telemonitoring and standard care groups decreased post-study (*P* = .001, *P* = .002, respectively). Similarly, NYHA class, LVEF, self-care maintenance, and self-care management improved for both groups. However, quality of life as measured with the MLHFQ significantly improved only for the telemonitoring group (*P* = .02), including the physical (*P* = .02) and emotional dimensions (*P* = .03).

A comparison of the post-study data between groups found only a statistically significant difference in SCHFI maintenance scores, indicating the telemonitoring group had greater self-care maintenance (ie, a higher SCHFI maintenance score) (*P* = .03). A comparison of the change scores between groups found only a statistically significant difference in the overall MLHFQ scores, indicating that the telemonitoring group had greater improvement in quality of life (ie, a larger difference from baseline to post-study MLHFQ scores) (*P* = .05).

**Table 3:** Results for BNP, NYHA class, LVEF, SCHFI, and MLHFQ scores

Parameter	telemonitoring group				standard care group				Between-group post-study data *P* value	Between-group change scores *P* value
	N	Baseline mean (SD)	Post-study mean (SD)	*P* value	N	Baseline mean (SD)	Post-study mean (SD)	*P* value		
BNP (pg/mL)	44	592 (538)	414 (604)	.001	44	426 (501)	303 (460)	.002	.2	.5
NYHA class	43	2.5 (0.6)	2.1 (0.7)	.000	38	2.6 (0.6)	2.2 (0.7)	.001	.8	.8
LVEF (%)	41	25.2 (8.8)	32.7 (11.8)	.001	35	24.8 (9.7)	31.3 (12.5)	.001	.7	.7
Self-care maintenance (SCHFI)	38	65.1 (18.6)	73.3 (11.6)	.004	44	58.9 (18.7)	65.5 (15.8)	.006	.03	.6
Self-care management (SCHFI)	18	58.1 (24.5)	68.6 (16.0)	.02	21	57.9 (22.4)	69.3 (18.3)	.01	.7	.9
Self-care confidence (SCHFI)	37	57.4 (20.6)	57.7 (19.5)	.7	43	55.8 (20.0)	56.2 (21.8)	.9	.9	.8
Quality of life (MLHFQ)	38	50.3 (29.1)	41.4 (26.7)	.02	44	47.8 (22.6)	47.3 (23.4)	.9	.2	.05
Quality of life – physical (MLHFQ)	38	21.7 (12.8)	17.8 (12.9)	.02	44	21.0 (10.5)	20.2 (10.5)	.5	.3	.1
Quality of life – emotional (MLHFQ)	38	11.7 (8.6)	9.5 (7.8)	.03	44	11.0 (7.0)	11.3 (6.9)	.8	.2	.07

As with previous studies of multidisciplinary heart function clinics, it was hypothesized being enrolled in the Heart Function Clinic would improve outcomes particularly for those patients new to the clinic. To test if there was a clinic effect on the outcome measures showing improvements in both groups, the changes in outcome measures were compared for patients who were new to the clinic (< 6 months, N = 37) versus those who were long-term patients (> 6 months, N = 63). We chose six months as the cut-off point because patients newly referred to the clinic usually require three months to up-titrate medication and a further three months to reach clinical stability. In the telemonitoring group, 18 patients were new to the clinic versus 19 in the standard care group. Patients who were new to the clinic improved more than those who were long-term with respect to BNP and LVEF (Table 4). Self-care maintenance of the new patients also improved more, but the difference was not statistically significant.

**Table 4:** Comparison of patients new to the Heart Function Clinic (enrolled < 6 months) and long-term patients (enrolled > 6 months)

Parameter	Patients enrolled < 6 months		Patients enrolled > 6 months		*P* value
	N	Baseline mean (SD)	N	Baseline mean (SD)	
Change in BNP (pg/mL)	35	279.8 (606.4)	53	65.4 (325.5)	.003
Change in NYHA class	33	0.5 (0.8)	44	0.4 (0.5)	.6
Change in LVEF (%)	34	−9.5 (10.6)	42	−5.1 (11.4)	.02
Change in self-care maintenance (SCHFI)	31	−10.6 (17.9)	51	−5.4 (14.1)	.1
Change in self-care management (SCHFI)	12	−9.5 (17.1)	27	−11.7 (19.4)	.7

To minimize the clinic effect, the statistical analysis was repeated post-hoc removing the data from the 37 patients new to clinic. Table 5 shows the subgroup statistical results for the parameters that improved for both groups. For long-term patients, the BNP (*P* = .02), LVEF (*P* = .005), self-care maintenance (*P* = .05), and self-care management (*P* = .03) significantly improved only for the telemonitoring group. A comparison of the post-study data and change scores between groups found only a statistically significant difference in post-study MLHFQ emotional dimension scores, indicating the telemonitoring group had better post-study quality of life (emotional dimension) compared to the standard care group (*P* = .05).

**Table 5:** Results for BNP, NYHA class, LVEF, and SCHFI scores with new patients to the clinic removed

Parameter	telemonitoring group				standard care group				Between-group post-study data *P* value	Between- group change scores *P* value
	N	Baseline mean (SD)	Post-study mean (SD)	*P* value	N	Baseline mean (SD)	Post-study mean (SD)	*P* value		
BNP (pg/mL)	27	583 (464)	433 (445)	.02	26	326 (296)	349 (467)	.4	.3	.1
NYHA Class	26	2.6 (0.5)	2.2 (0.7)	.002	18	2.7 (0.6)	2.3 (0.5)	.01	1.0	.9
LVEF (%)	24	23.9 (8.9)	31.3 (12.3)	.005	18	28.1 (10.5)	30.1 (14.7)	.4	.9	.1
Self-care maintenance (SCHFI)	23	64.9 (20.3)	71.9 (12.7)	.05	28	59.1 (18.7)	63.1 (16.9)	.1	.07	.5
Self-care management (SCHFI)	13	51.5 (23.5)	65.0 (12.9)	.03	14	62.1 (17.8)	72.1 (12.0)	.08	.2	.7

### Mortality Results

During the trial, three patients from the telemonitoring group died and none died from the standard care group. One patient died from newly diagnosed cancer, the second was suspected to have died from sepsis due to a leg ulcer, and the third died from post-heart transplantation complications (transplant performed prior to study enrollment).

### Health Care Resource Utilization Results

Table 6 shows the results from the Mann–Whitney tests comparing the health care resource utilization by the telemonitoring and standard care groups during the trial. No differences were found between the groups for number of hospitalizations (*P* = .1), number of nights in hospital (*P* = .2), or number of visits to the emergency room (*P* = .6). However, the telemonitoring group visited the Heart Function Clinic during the six months more often than the standard care group (*P* = .04) because the cardiologist managing the alerts asked several patients to come into clinic when their health appeared to be deteriorating, as alerted by the telemonitoring system.

**Table 6:** Results for health care resource utilization

Parameter	telemonitoring group		standard care group		*P* value
	N	Mean (SD)	N	Mean (SD)	
Number of hospital admissions	38	0.5 (0.8)	44	0.2 (0.4)	.1
Number of nights in hospital	38	2.3 (5.3)	44	1.3 (4.2)	.2
Number of emergency department visits	38	0.4 (0.9)	44	0.3 (0.5)	.6
Number of Heart Function Clinic visits	39	3.5 (3.6)	45	2.5 (2.5)	.04

### Medication and Blood Test Results

Table 7 shows the number of patients prescribed angiotensin-converting enzyme (ACE) inhibitor and/or angiotensin receptor blocker (ARB), beta-blocker, diuretic, statin, aldosterone antagonist, digoxin, and antiarrhythmic medication at baseline and post-study for the telemonitoring and standard care groups. Using McNemar’s test, no statistical differences were found between the frequency of medication prescribed at baseline compared to the frequency of medication prescribed post-study for either of the groups, except more of the patients in the telemonitoring group were prescribed aldosterone antagonist post-study (*P* = .02). Seven patients in the telemonitoring group started to take aldosterone antagonist during the six-month trial. No significant statistical differences were found comparing the post-study frequency of medication between groups using Fisher’s exact test. No significant differences were found between baseline and post-study creatinine, sodium, potassium, hemoglobin, and urate blood test results.

**Table 7:** Number of patients prescribed various types of medication

Medication	telemonitoring group					standard care group					Between-group post-study data *P* value
	Baseline		Post study		*P* value	Baseline		Post study		*P* value	
	Prescribed	Not prescribed	Prescribed	Not prescribed		Prescribed	Not prescribed	Prescribed	Not prescribed		
ACE-inhibitor and/or ARB	49	1	44	1	1.0	48	2	36	2	1.0	.6
Beta-blocker	49	1	44	1	1.0	49	1	38	0	1.0	1.0
Diuretic	47	3	42	3	1.0	45	5	34	4	1.0	.7
Statin	31	19	27	18	1.0	26	24	22	16	.3	1.0
Aldosterone antagonist	23	27	27	18	.02	29	21	23	15	.7	1.0
Digoxin	16	34	16	29	1.0	22	28	16	22	.7	.7
Antiarrhythmic	14	36	12	33	1.0	6	44	6	32	1.0	.3

## Discussion

A randomized controlled trial was performed to evaluate a user-centered, mobile phone-based telemonitoring system. Although the trial was underpowered to detect its impact on hospitalization and mortality, the results suggest quality of life improved with the use of the system through increased self-care and improved clinical management. BNP, LVEF, and NYHA class all improved over the course of the trial for both the telemonitoring and standard care groups. A subgroup analysis using only the participants who had attended the clinic for more than 6 months showed only the telemonitoring group had significant improvements in BNP and LVEF from baseline to post-study. It is possible a trial with a larger sample size would find a reduction in hospitalization and mortality in the telemonitoring group.

One of the most significant changes in clinical management with the telemonitoring system was the ability to optimize a patient’s medication regimen. For example, there was a statistically significant increase in the number of patients in the telemonitoring group who were prescribed aldosterone antagonist compared to the standard care group. Previous studies have found less than a third of eligible patients receive heart failure guideline-recommended aldosterone antagonist therapy [15]. The benefits of aldosterone antagonist, in terms of reductions in mortality and hospitalizations, have been well documented. However, patients are often not prescribed this therapy partially because of the need to closely monitor serum potassium levels due to the risk of hyperkalemia [16]. It is possible the increase in use of aldosterone antagonist in the telemonitoring group can be attributed to the close monitoring of the patients enabled by the telemonitoring system.

In terms of self-care, the telemonitoring system provided immediate automated instructions and enabled clinical intervention at the most appropriate time (“teachable moments”) to help patients modify their lifestyle behaviors. For example, many patients found their weight and blood pressure increased after a high sodium meal. By reducing their salt intake for the next few meals, their weight and blood pressure would return to within their normal range. Some patients also received automated reminders to take extra diuretic medication after a weight gain, which confirmed taking the extra medication was the correct course of action. Even though this group of patients received prior instruction from their cardiologist to take extra diuretic medication in this situation, they were still often hesitant to take the extra medication without prior confirmation.

A large-scale (N=1653) randomized controlled trial, Telemonitoring to Improve Heart Failure Outcomes (Tele-HF), was conducted using as a primary outcome measure a composite of readmission for any reason or death from any cause within 180 days of enrollment [17]. That trial found no reduction in mortality or hospital admissions from the use of a telephone-based interactive voice-response system to record heart failure symptoms and weight data. One of the possible reasons no differences were found between groups is the Tele-HF trial attempted to engage patients in self-care only in terms of performing the daily reporting of symptoms and weight. Real-time automated self-care advice and instructions (taking extra diuretic medication, following salt and fluid restrictions, etc) based on the reported symptoms and weight—as implemented in our study—might have had a significant positive impact. In addition, site coordinators reviewed the patient information daily on weekdays for the Tele-HF trial and should have contacted the patient as required. It is possible the necessary real-time response by clinicians was not provided.

Finally, 14% of the Tele-HF patients randomized into the intervention group never used the system, and only 55% of the patients were using the system at least 3 times per week by the final week. The Tele-HF trial lasted 180 days, which was similar to our trial. However, by the final week of our trial, 89% of our patients were taking their measurements at least 3 times per week (excluding the 3 patients who had died). Our high rate of adherence may be due to the perceived benefit, ongoing positive reinforcement, and ease of use of our system even among the very elderly (the oldest patient in the telemonitoring group was aged 88 years). The trial by Scherr et al (2009) also found low adherence rates due to patients having difficulty in manually entering and sending their daily blood pressure, heart rate, body weight, and dosage of heart failure medication through a mobile phone’s Internet browser [6]. The differences in adherence rates between these trials underscore the importance of system design on the successful implementation of telemonitoring systems.

The TIM-HF trial investigated the effects of wireless medical devices with a personal digital assistant (PDA) on health outcomes of 710 heart failure patients randomized into intervention and control groups. Similar to the Tele-HF trial, this trial found no statistically significant differences between the groups with respect to mortality, number of hospitalizations, or days in hospital [5]. However, an exploratory subgroup analysis suggested the efficacy of telemonitoring to improve heart failure outcomes could be dependent on the characteristics of the patient population. The investigators concluded further trials were required to explore the effects of telemonitoring in defined patient subgroups.

In our study, being newly enrolled into the Heart Function Clinic overshadowed improvements from the telemonitoring system in terms of the within-group analysis. In addition, many of our participants had stable heart failure, and had not been admitted to hospital for many years. Bowles et al (2009) also suggested targeting higher risk patients with greater probability of being rehospitalized in order to demonstrate the most effective outcomes from telemonitoring [18]. Eligibility criteria for study participation that included admission to hospital within the previous year and being part of the Heart Function Clinic for at least 6 months may have resulted in more significant improvements in health outcomes.

Although the clinicians viewed the ECG recordings that were available, the benefits of the ECG recordings compared to the costs of the devices were not conclusive. Feedback from the clinicians indicated they thought the ECG recordings were of some use, but the inability to provide the devices to participants with ICDs was a significant drawback.

### Limitations

A confounder to our study was the clinic effect that caused improvements in outcomes in both the telemonitoring and standard care groups, as described above. Future studies should consider recruitment of stable clinic patients. Secondly, patients were enrolled in the winter and completed the trial in the summer when heart failure patients are often healthier due to reductions in respiratory illnesses. This seasonal effect may have also contributed to the observed improvements in both the telemonitoring and standard care groups. A third limitation was the small sample size that did not provide adequate power to detect the effects of the telemonitoring on mortality and hospitalization outcomes. A future trial with a larger sample size would help further determine the effects of the telemonitoring system on health service utilization and its health economic impact. In addition, the proportionate benefit of self-care versus clinical management changes on health outcomes could not be definitively determined. For example, the improvements observed in the telemonitoring group may be largely attributed to the increased prescription of aldosterone antagonist. Furthermore, about a third of the patients in the telemonitoring group used the telemonitoring system for a number of weeks prior to completing the baseline questionnaire. Although the patients were instructed to answer the questionnaire based on information before they used the telemonitoring system, it was clear many patients were basing their answers on information associated with system use because their questionnaire responses sometimes did not match the information provided during their recruitment interviews. This minimized the measured impact of the telemonitoring system. Limitations to the questionnaire data also include potential recall bias and self-reporting. Finally, the telemonitoring system was only available in English, which limited the participant population to patients who were able to read rudimentary English.

### Conclusions

The results from our trial suggest mobile phone-based telemonitoring improves quality of life through improved self-care and clinical management. A subgroup analysis using only the participants who had attended clinic for more than 6 months showed only the telemonitoring group had significant improvements in BNP and LVEF from baseline to post-study. An important component to successful telemonitoring for heart failure appears to be immediate feedback to the patients to address any potential decompensation either through automated messages and/or advice from a clinician who is familiar with patients’ histories. In addition, in order for patients to be willing to integrate telemonitoring into their daily lives, the system must be easy and quick to use. Further research with an appropriate heart failure patient population and larger sample size is required to determine the extent of the benefits of such a telemonitoring system on heart failure outcomes.

## Abbreviations

ACE: angiotensin-converting enzyme
ARB: angiotensin receptor blocker
BNP: brain natriuretic peptide
ECG: electrocardiograms
ICD: implantable cardioverter defibrillator
LVEF: left ventricular ejection fraction
MLHFQ: Minnesota Living with Heart Failure Questionnaire
NYHA: New York Heart Association
RCT: randomized controlled trial
SC: standard care
SCHFI: Self-Care of Heart Failure Index
SD: standard deviation
Tele-HF: Telemonitoring to Improve Heart Failure Outcomes
TIM-HF: Telemedical Interventional Monitoring in Heart Failure
telemonitoring: telemonitoring
UHN: University Health Network
